# Tanzania’s and Germany’s Digital Health Strategies and Their Consistency With the World Health Organization’s Global Strategy on Digital Health 2020-2025: Comparative Policy Analysis

**DOI:** 10.2196/52150

**Published:** 2024-03-18

**Authors:** Felix Holl, Jennifer Kircher, Attila J Hertelendy, Felix Sukums, Walter Swoboda

**Affiliations:** 1 DigiHealth Institute Neu-Ulm University of Applied Sciences Neu-Ulm Germany; 2 Department of Information Systems and Business Analytics, College of Business Florida International University Miami, FL United States; 3 MUHAS Digital Health and Innovation Research Group Muhimbili University of Health & Allied Sciences Dar es Salaam United Republic of Tanzania

**Keywords:** digital health strategies, comparative policy analysis, DHS, eHealth, digital health, strategy, strategies, policy, policies, document analysis, document analyses, GSDH, Germany, Europe, Africa, Tanzania

## Abstract

**Background:**

In recent years, the fast-paced adoption of digital health (DH) technologies has transformed health care delivery. However, this rapid evolution has also led to challenges such as uncoordinated development and information silos, impeding effective health care integration. Recognizing these challenges, nations have developed digital health strategies (DHSs), aligning with their national health priorities and guidance from global frameworks. The World Health Organization (WHO)’s Global Strategy on Digital Health 2020-2025 (GSDH) guides national DHSs.

**Objective:**

This study analyzes the DHSs of Tanzania and Germany as case studies and assesses their alignment with the GSDH and identifies strengths, shortcomings, and areas for improvement.

**Methods:**

A comparative policy analysis was conducted, focusing on the DHSs of Tanzania and Germany as case studies, selected for their contrasting health care systems and cooperative history. The analysis involved a three-step process: (1) assessing consistency with the GSDH, (2) comparing similarities and differences, and (3) evaluating the incorporation of emergent technologies. Primary data sources included national eHealth policy documents and related legislation.

**Results:**

Both Germany’s and Tanzania’s DHSs align significantly with the WHO’s GSDH, incorporating most of its 35 elements, but each missing 5 distinct elements. Specifically, Tanzania’s DHS lacks in areas such as knowledge management and capacity building for leaders, while Germany’s strategy falls short in engaging health care service providers and beneficiaries in development phases and promoting health equity. Both countries, however, excel in other aspects like collaboration, knowledge transfer, and advancing national DHSs, reflecting their commitment to enhancing DH infrastructures. The high ratings of both countries on the Global Digital Health Monitor underscore their substantial progress in DH, although challenges persist in adopting the rapidly advancing technologies and in the need for more inclusive and comprehensive strategies.

**Conclusions:**

This study reveals that both Tanzania and Germany have made significant strides in aligning their DHSs with the WHO’s GSDH. However, the rapid evolution of technologies like artificial intelligence and machine learning presents challenges in keeping strategies up-to-date. This study recommends the development of more comprehensive, inclusive strategies and regular revisions to align with emerging technologies and needs. The research underscores the importance of context-specific adaptations in DHSs and highlights the need for broader, strategic guidelines to direct the future development of the DH ecosystem. The WHO’s GSDH serves as a crucial blueprint for national DHSs. This comparative analysis demonstrates the value and challenges of aligning national strategies with global guidelines. Both Tanzania and Germany offer valuable insights into developing and implementing effective DHSs, highlighting the importance of continuous adaptation and context-specific considerations. Future policy assessments require in-depth knowledge of the country’s health care needs and structure, supplemented by stakeholder input for a comprehensive evaluation.

## Introduction

Technological advances have fostered the adoption of new digital health (DH) technologies [1]. Such adoption has been so rapid that negative side effects such as uncoordinated implementation have resulted [2]. Uncoordinated development can lead to siloed information systems with limited interpretability with other information systems [3]. To combat uncoordinated development, countries have developed digital health strategies (DHSs) to set strategic guidance for the future development of DH technologies, often aligned with their own national health priorities. The World Health Organization (WHO) developed the Global Strategy on Digital Health 2020-2025 (GSDH) to provide global guidance to member countries in defining their DHSs [4]. It is the first comprehensive strategy for DH globally [5]. The GSDH encourages the development of national DHSs based on expert knowledge and WHO member state consensus. National DHSs often fail to capture the most emergent technologies such as artificial intelligence (AI), machine learning, or drones because of their fast development cycles, which can lead to uncoordinated development and adoption [6]. While no country has reported that they used the GSDH specifically to develop their national DHS yet, the GSDH may have informed elements of national DHS and existing DHSs can be assessed for their consistency with the GSDH.

Tanzania’s Ministry of Health recognizes the importance of DH technologies in health care delivery and has published 2 strategy documents within the last decade. The *Tanzania National eHealth Strategy 2013-2018* focused on the infrastructure and technologies needed to support the transformation of the health sector [7]. In contrast, the latest *Digital Health Strategy July 2019-June 2024* addresses, among other aspects, challenges that have not been addressed or solved and the best use of technology to improve patient care [8]. It also focuses on strategic priorities aligned with the WHO health system building blocks and advocating user-centric, interoperable, and data-driven DH interventions.

Germany has a federal system of government, with 16 state-level ministries of health and 1 Federal Ministry of Health (FMoH) plus other institutions responsible for different areas of DH. Between 2015 and 2021, each entity published corresponding strategies, strategic documents, recommendations, or legislation about the digitalization of health care in Germany, many of which were included in this study. Before the publication of its first National Digitalization Strategy in March 2023 [9], Germany lacked a uniform strategy with binding goals and guidelines.

Tanzania and Germany have a history of cooperation in socioeconomic development, including health sector improvement. The 2 countries were chosen as case studies of the development of DHS in the global South and North. We draw on our own extensive experience in developing and implementing DH systems in these 2 countries. The purpose of this study is to investigate the key elements and possible shortcomings of the DHSs of Tanzania and Germany, investigate their alignment with the WHO GSDH, and make applicable recommendations to improve the DHS for the 2 countries. We also aim to identify aspects of the WHO GSDH that are challenging for countries to implement. The comparison of the WHO GSDH with the DHSs of Tanzania and Germany is intended as a case study for this policy comparison approach to conduct additional analyses and share this method with other researchers to stimulate similar analyses in other countries.

## Methods

We compared the DHSs of Tanzania and Germany and assessed their consistency with the WHO’s GSDH, using a document analysis and comparative policy analysis approach [10,11]. We selected Tanzania and Germany as case studies for this comparison, as the researchers had in-depth knowledge about the health care systems and the state of DH in the 2 countries. This in-depth understanding is a requirement for case studies in comparative policy analyses [11]. We searched for the relevant primary data sources through searches in academic databases, through search engines, and based on the expert knowledge of the researchers and other subject matter experts. We identified primary data sources that were national eHealth policy documents and technology and health-related policy documents (see Textbox 1).

**Primary data sources from the World Health Organization, Tanzania, and Germany used for the analysis with references.**

Global Strategy on Digital Health 2020-2025—World Health Organization [4]

Digital Health Strategy July 2019-June 2024—Tanzania [8]

Different Approaches of Digital Health Strategies—Germany (various sources, a list of all included laws and regulations can be found in the Results section)

Once the primary data sources were identified, data extraction and analysis were done in six steps: (1) development of a category system for data extraction and analysis; (2) data extraction; (3) assessment of the data from Germany and Tanzania for their compliance with the GSDH; (4) analysis of similarities and differences between of the strategies of Germany and Tanzania; (5) categorical summary and regrouping into policy, infrastructural, and human factors; and (6) assessment of how new technologies are incorporated in each strategy.

In the first step of this study, we created a deductive category system to assess consistency with the WHO’s GSDH 2020-2025. This step was conducted by 1 researcher (JK) and validated by a second researcher (FH). The categories were derived from the 4 dimensions defined in the GSDH:

- collaboration and knowledge transfer
- advance the implementation of national DHSs
- strengthen governance for DH at global, regional, and national levels
- integrated people-centered health systems enabled by DH technologies

Data from the primary data sources were extracted and classified into 1 of the 4 categories by 1 researcher (JK). A second researcher (FH) validated both the extrication and the classification. After the initial classification, the results within each category were categorized into one of the three subsections: (1) policy options, (2) measures, or (3) outcomes. The initial categorization was done by 1 researcher (JK) and validated by a second researcher (FH).

After the extrication and categorization were completed, we assessed all extracted data for compliance with the GSDH (initial assessment by JK, validation for Germany by FH and for Tanzania by FS). In the second step of the analysis, we identified similarities and differences in the strategies (initial assessment by JK, validation for Germany by FH and for Tanzania by FS). Following an inductive content analysis (by JK), in a third step, the extracted data were summarized categorically and regrouped into *policy, infrastructural,* and *human factors* [12] (by JK, and validated by FH and FS). Finally, as the fourth and last step, the extent to which new technologies such as AI, machine learning, or drones were included in the DHSs was assessed (initial assessment by JK, validation for Germany by FH and for Tanzania by FS). A detailed breakdown of the results of the policy comparison by aspect can be found in the Multimedia Appendix 1.

### Ethical Considerations

This study only analyzed policy documents. No human subjects were investigated or personal data analyzed. All data that were handled as part of this study were stored and analyzed on encrypted devices. Any personal identifiers from the primary data, such as author names or contact details were removed during data extraction of the primary data into our data set. The extracted data are available in a summarized form in the Multimedia Appendix 2 [4,7-9,13-27] and the full data set is available on request from the authors.

## Results

### Consistency With the WHO’s GSDH 2020-2025

Both Germany’s and Tanzania’s DHS include most of the 35 elements from the WHO’s GSDH. Both DHSs do not include 5 elements each. The items that are not included are shown in Table 1.

**Table 1:** Items of the World Health Organization’s GSDH^a^ not included in the DHSs^b^ of Tanzania and Germany

Section		Item
**Tanzania**		
	(1) Collaboration and knowledge transfer: policy options and actions	(2) Establish a knowledge management approach to identify and share good practices, knowledge about the implementation of new methods and techniques, evidence, and lessons learned on digital health across countries and international communities
	(3) Strengthen governance for digital health at global, regional, and national levels: policy options and actions	(4) Promote capacity-building for leaders of public health authorities and affiliated agencies, and policy makers to make informed decisions to support digital health investments
	(3) Strengthen governance for digital health at global, regional, and national levels: outputs	(3) Global guidance on planning, development, and use of digital hospitals, digital clinical trials, and digital therapeutics is developed
	(3) Strengthen governance for digital health at global, regional, and national levels: outputs	(4) A set of recommendations is developed for pseudonymization and anonymization of health data
	(4) Advocate people-centered health systems that are enabled by digital health: outputs	(4) Global guidance on personalized medicine is developed
**Germany**		
	(2) Advance the implementation of national DHSs: policy options and actions	(2) Ensure that institutions, decision makers, and personnel involved in the provision of health care services and all end user communities and beneficiary populations are adequately engaged in the design and development phases
	(2) Advance the implementation of national DHSs: policy options and actions	(7) Design, implement, and monitor a change management plan, to support conducive organizational behavior surrounding newly digitized health processes and practices
	(3) Strengthen governance for digital health at global, regional, and national levels: policy options and actions	(4) Promote capacity-building for leaders of public health authorities, affiliated agencies, and policy makers to make informed decisions to support digital health investments
	(4) Advocate people-centered health systems that are enabled by digital health: policy options and actions	(4) Strengthen gender equality and health equity approaches and accessibility for people with disabilities to promote an inclusive digital society with enhanced digital health skills. When planning and prioritizing digital health interventions, relevant factors of inequality should be assessed to ensure that the introduction of digital health technologies does not aggravate these (“do no harm”) and that access for specific population groups is guaranteed. In addition, the specific potential of digital technologies to promote health equity should be leveraged. Designed properly, digital solutions can propel inclusiveness as digital connectivity can transcend physical barriers
	(4) Advocate people-centered health systems that are enabled by digital health: outputs	(2) A framework allowing individual feedback in validating the performance of digital health tools and services, diffusion of increasing digital health demand is implemented and used

^a^GSDH: Global Strategy on Digital Health 2020-2025.

^b^DHS: digital health strategy.

### (1) Collaboration and Knowledge Transfer

As a member of the European Union, Germany is involved in shaping a global DHS and published the strategy document *Strategy of the Federal Government on Global Health* in 2020 [13]. Germany participates in various programs such as Horizon Europe, aiming to promote a knowledge- and innovation-based society and a competitive, sustainable economy [14]. Multi-stakeholder meetings are convened to overcome the implementation hurdles of digitale gesundheitsanwendungen (DiGAs) and innovations through various initiatives. For example, the goal of the *German Alliance for Global Health* *Research* is to expand its research network [15]. To facilitate the exchange of results with partners and institutions across countries, the focus of the *German Alliance for Global Health* *Research* is also on a research-compatible data infrastructure with international standards [16]. Knowledge transfer, especially to the global south is an integral part of the German strategy.

Tanzania’s strategy focuses primarily on national challenges and opportunities. The aim is to improve health services at all levels of the country’s health system [8]. Key stakeholders are assigned to sectors in the strategy [8]. According to priority 3 of Tanzania’s strategy, a knowledge management approach will be expanded and developed only at the population and health worker level, for example, through e-learning platforms [8]. More digital solutions are being developed, according to priority 10 [8], to improve surveillance of and reporting on notifiable diseases, disease outbreaks, and disasters to prevent loss of life and socioeconomic impact [28].

### (2) Advance the Implementation of National DHSs

Due to the prevailing federalism in Germany, each federal state has its own Ministry of Health at the state level and can therefore enact its own laws and rules. However, higher-level legislation such as the *E-Health Law* [17], which was introduced in 2015, can force the federal states to adapt to the national strategy. The E-Health Law has set the initial course for the development of the secure telematics infrastructure (TI) and the introduction of DiGAs. The law is driving forward digitalization in the health care sector for the benefit of patients. It contains a roadmap for the introduction of a digital infrastructure and allows insured persons to benefit from specific applications [17]. In addition, at annual conferences, the health ministers of all federal states represent and discuss the interests of the federal states and health policy issues [18]. Instead of a national digitization strategy, Germany has formal roadmaps based on legislation and recommendations, such as the *Roadmap Digital Health* [19]. Within the framework of the Innovation Forum *Digital Health 2025* of the FMoH*,* 5 fields of action for the future have been defined, which function as a target and implementation blueprint. The five fields are (1) building a sustainable basis, (2) digital care as the normality, (3) overcoming institutional and sectoral (digital) boundaries, (4) strengthening data literacy—making health data usable, and (5) using new technologies to enable individualized medicine [16]. However, a clear and structured prioritization to achieve the goals is not evident.

To lead Tanzania into the digital age and guide progress and development, the government developed the *Digital Health Strategy July 2019-June 2024*. This strategy includes a clear prioritization derived from the *National Health Policy 2019* and complemented by a rigorous consultation process with key stakeholders in the health sector [8]. It sets standards for data and technologies and strengthens interoperability between systems and sectors [8]. “Improving the legal and regulatory framework to ensure client safety, data security, confidentiality, and privacy” is identified in priority 1 [8]. It identifies the development of a change management plan as an essential factor for the successful implementation and adoption of DH solutions.

### (3) Strengthen Governance for DH at Global, Regional, and National Levels

In Germany, several laws and regulations came into force in 2019 and the following years, leading to a stable legal framework for DH. Most recently, the *Health IT Interoperability Governance Regulation* was published in 2021, specifying the tasks of the interoperability coordination unit and the expert panel [20]. Other programs, such as the innovation initiative “Data for Health: Roadmap for Better Patient Care through Health Research and Digitalization” [21], and various working groups, deal with data context [22]. DiGAs are described as particularly innovative and are defined as prescription applications reimbursable by the public health insurance system [29]. As such, DiGAs must meet high requirements and demonstrate an evidence-based medical benefit. All these laws and regulations were related to DH. An overview of them can be found in Table 2.

**Table 2:** Laws and regulations regarding digital health in Germany with their respective year of publication.

Reference	Law and regulation	Year of publication
[9]	DIGITAL TOGETHER Germany’s Digitalisation Strategy for Health and Care	2023
[13]	Strategie der Bundesregierung zur globalen Gesundheit	2020
[14]	Horizon Europe Program	2021
[16]	Digitale Gesundheit 2025	2020
[17]	Gesetz für sichere digitale Kommunikation und Anwendungen im Gesundheitssektor	2015
[19]	Roadmap Digitale Gesundheit	2018
[20]	Gesundheits-IT Interoperabilität Governance Verordnung	2021
[21]	Daten helfen heilen	2020
[22]	Datenschutz und IT-Sicherheit im Gesundheitssektor	2022
[23]	Gesetz zur digitalen Modernisierung von Versorgung und Pflege (Digitale-Versorgung-und-Pflege-Modernisierungs-Gesetz-DVPMG)	2022
[24]	Hightech-Strategie 2025	2021
[25]	European Health Data Space	2022

For Tanzania, Digital Health Strategy July 2019-June 2024 identifies responsibilities and organizations for a sustainable governance structure and specified projects for developing guidelines for implementation and creating a legal and regulatory framework for DH under priority 1 [8]. According to priority 6, the aim is to develop analytical tools and indicators with the data and use them for evidence-based interventions and decision-making [8].

Tanzania explicitly mentions strengthening programs for continuous professional development of health workers in the use of data. Priority 6 is to include aspects of data use in health care worker educational and professional development curricula [8]. In addition, there is a focus on networking health professionals by introducing digital platforms such as e-learning (priority 3) [8].

### (4) Integrated People-Centered Health Systems Enabled by DH Technologies

The German federal government wants to promote patient autonomy. Implementing a national electronic patient file enables patients to retain complete control over their data and retain decision-making authority over their medical records [30]. To promote accessibility to health tools, a national health portal was created [31]*.* In addition, the German government wants to promote personalized treatment approaches in all important disease categories and foster early cooperation between stakeholders from science, industry, regulatory authorities, and the medical and patient communities [32]. The *Digital Care and Nursing Modernisation Act* stipulates that, in the future, more DiGAs, digital nursing applications, and telemedical applications should support doctors and nurses and help them perform their tasks more efficiently [23]. To enable the government to monitor the current state of digitization, the gematik (gematik GmbH) has developed a dashboard of key performance indicators that tracks the TI in Germany. Further, gematik is the public entity tasked with developing and maintaining the TI in the country [33]. Germany has not adopted formal strategic approaches to strengthening gender equality and inclusion in the context of digitalization.

Tanzania’s DHS focuses heavily on using digital technologies and improving DH competencies. The aim of priority 3 is to provide specialized care to underserved facilities using digital technologies and train health workers with the appropriate skills [8]. The objective is to improve health care facility processes through digital solutions, such as electronic referrals, which also helps to relieve the demand on staff resources (priority 2) [8]. Health information should be shared and disseminated using mobile health, short messaging services, mobile apps, and web apps, thus contributing to patient education (priority 4) [8]. For the government to assess and improve the quality of health services efficiently, priority 6 is to introduce digital solutions for monitoring facilities [8]. Priority 8 also describes measures using digital tools to monitor human resources [8]. However, approaches to strengthen gender equality and accessibility for people with disabilities to promote an inclusive digital society are not addressed.

### Similarities and Differences in the Strategies

Our evaluation system is based on the degree to which the policy options, measures, and outputs of each country fulfill the respective dimensions of the WHO strategy from today’s perspective. The 4 rating options are 0=not present, 1=partly fulfilled, 2=largely fulfilled, and 3=completely fulfilled. Multimedia Appendix 3 illustrates the results. The analysis highlights Tanzania’s plans to strengthen national health policy through targeted implementation measures in the strategy. On the other hand, Germany fulfills all the points recommended by the WHO by expanding its cross-standard data infrastructure. While both countries consider a people-centered health system to be highly relevant, we nevertheless rate the current measures as just above average due to, for example, the lack of gender equality and inclusion concepts in the context of digitalization in the strategies. Strengthening governance for DH at global, regional, and national levels is only partially met in both strategies. In particular, the development of leadership for informed decision-making and the expansion of the strategy to a global perspective limit the fulfillment of the dimension. There is an overall similarity between both countries in terms of their overall progress on their respective DHS. Individual different focus points have therefore already been identified.

In the following, the extracted data were summarized categorically and regrouped into *policy, infrastructural*, and *human factors*.

### Policy Factors

The WHO GSDH includes a vision, strategic goals, a framework for action, and principles for implementation to advance DH globally and at the state level. Until March 2023, Germany did not develop a national digitization strategy. Priority topics can be found in political documents such as Digital Health 2025. Although most elements of the WHO are mentioned, various aspects are weighted differently. Germany focuses on overcoming sectoral care boundaries and developing innovative technologies such as AI.

While Germany uses national legislation to create a comprehensive legal framework aligned with its national health system, Tanzania has already established governance structures and is now focusing on building the capacity of new members and stakeholders at lower levels. Tanzania’s national strategy is based on a vision, associated goals, and clearly articulated priorities. Among these strategic priorities are all 4 main key points identified in the WHO GSDH. Tanzania also complements the WHO GSDH and strives, among other things, to improve supply chain management of health commodities and to improve human resource management at all levels of the health system. Compared to Germany, Tanzania’s strategy details developing a change management plan.

### Infrastructure Factors

The WHO aims to promote international cooperation by intensifying knowledge transfer among member states. As part of the European Union, Germany pools its resources for international projects and programs and supports DH globally. One of the goals is to create a research-compatible data infrastructure to strengthen the interoperability of systems following international data exchange standards. In Germany, the gematik dashboard is used as an assessment tool to make an initial assessment of the maturity of digital solutions. As part of the European Union, Germany has an overarching basic regulation in the form of the General Data Protection Regulation [33].

Tanzania has also developed the *Tanzania Health Enterprise Architecture,* an approach developed to simplify the complexity of health information systems, guide the development of DH solutions, and facilitate system interoperability [26]. The Tanzanian government highly prioritizes safeguarding the security of sensitive personal data, such as medical information, and is dedicated to improving the legal and regulatory framework to ensure data security, confidentiality, and privacy protection.

### Human Factors

Both Tanzania and Germany aim to place patients at the center of their health care systems but take different approaches to achieve this end. Germany actively strives to integrate the patient into health care delivery processes. Germany also aims to intensify research into personalized medicine. Digital solutions such as DiGAs or digital nursing applications (digital applications to support nursing care) focus on providing relief to medical and nursing staff, for example, by supporting labor-intensive tasks such as medical documentation.

Tanzania plans to use client-centric technologies to respond to clients’ needs through user-centered design to ensure a responsive, resilient, and inclusive health system. In terms of health care staff, Tanzania is investing in education and training to improve the digital skills of its health care system staff. Only Tanzania’s DHS mentions the need to promote health care management staff briefly, and neither Tanzania’s nor Germany’s DHS defines such steps. Similarly, neither strategy addresses strengthening gender equality and inclusion in digitalization.

### Incorporation of New Technologies

New technologies and developments in the health care system can be used to overcome care bottlenecks by increasing efficiency and effectiveness and thus reduce overuse, underuse, and misuse of health care [34].

The WHO recommends introducing sustainable financing models to benefit from the opportunities, realize the full potential of these innovations, and support the exchange of knowledge [4].

### Tanzania

To strengthen public health services, the government of Tanzania has focused on developing and expanding information and communication technologies (ICTs). However, further investment is needed to harness the ICT infrastructure for effective data systems such as AI. The goal is to use the huge amounts of data already being collected today effectively and productively. In collaboration with development partners, the Tanzanian government is looking for ways to increase investment in and use of health data systems [27].

In a scoping review conducted by Sukums et al [35] in 2021, a total of 16 publications were identified to have explored the use of AI-driven solutions in Tanzania’s health sector. The review called for Tanzania to establish a national AI policy and a regulatory framework for adopting reliable AI solutions in the health sector in line with the WHO guidance on ethics and governance of AI for health.

### Germany

The German federal government’s *Hightech-Strategy 2025,* creates a basis for investing in innovation by providing incentives for investment [24]. The federal government thus supports local health structures with direct funding and promotes synergies with the private sector and health promotion and disease prevention approaches.

The Federal Ministry of Education and Research has allocated around 250 million euros from 2018 to 2025 for therapy and care concepts implementing AI [36]. The Innovation Committee of the Joint Federal Committee also continuously promotes new technologies used in innovative forms of health care provision [37]. There are many different models for funding new technologies in Germany, many of which involve limited and temporary funding. A sustainable strategy or roadmap for developing and implementing innovative solutions is lacking.

### Global Digital Health Monitor

We also retrieved the assessments for Tanzania and Germany from the Global Digital Health Monitor [38]. Tanzania received a rating in terms of an overall DH phase of 4 (out of 5, with 1 being the lowest phase and 5 the highest) and Germany received a 5. For Germany, 4 out of 7 categories were not assessable. Scores for all categories are displayed in Table 3.

**Table 2:** Ratings on the Global Digital Health Monitor for Tanzania and Germany.

Characteristics	Tanzania	Germany
Leadership and governance	5	5
Strategy and investment	4	N/A^a^
Legislation, policy, and compliance	4	5
Workforce	2	N/A
Standards and interoperability	5	N/A
Infrastructure	4	5
Service and applications	4	N/A
Overall digital health phase	4	5

^a^N/A: not applicable.

## Discussion

### Principal Findings

Both Germany’s and Tanzania’s DHS include most of the 35 elements from the WHO’s GSDH. While both DHSs fail to include 5 of the 35 elements, they each include additional aspects not included in the GSDH that relate to their own country-specific health care system challenges. The Tanzanian DHS emphasizes (1) quality aspects and data use, (2) digital solutions for supply chains and resource optimization, and (3) human resources. These aspects are oriented toward national health priorities at all health system levels. In contrast, the German DHS focuses mainly on overcoming sectoral boundaries. Overall, both countries consider context in formulating goals and priorities. Both countries also receive high ratings on the Global Digital Health Monitor, with an overall DH phase rating of 4 out of 5 for Tanzania and 5 out of 5 for Germany. Tanzania has already developed its digitalization strategy for the health sector and identified tangible goals, priorities, and measures. In contrast, Germany does not have a unified strategy and is trying to cover a large distance by taking small steps. All the policies from Germany included in this analysis are from the federal level, meaning that even though Germany is a federal republic, the investigated strategies apply to the whole country.

Tanzania mainly uses new technologies to strengthen its health care system and improve the health and quality of life of its population through intensive health education of the population. Further, one requirement to achieving these goals is to move toward universal access to fiber optic cable and mobile. In recent years, the Tanzanian government has intensified the expansion of ICTs and focused intently on expanding the use of mobile health and telemedicine. It also recognizes the enormous potential of processing large data and has entered public-private funding partnerships to accelerate data infrastructure development and drive innovation. However, as evidenced by the not entirely successful use of machine learning to predict and stem cholera outbreaks, such practices still have weaknesses, and there is a lack of experts skilled in securely collecting, processing, and interpreting large data [35]. In its latest DHS, Tanzania commits to deepening the competencies of health care workers by intensifying e-learning, training, and new curricula. The Tanzanian government is actively trying to improve the health care situation in its country and is looking into sustainable financing models. Addressing the cost implications of DH is a key factor for future DHS development. Germany, meanwhile, is involved in helping to shape global health priority-setting and policy making. Its national ministries are promoting financing models for new technologies and innovations from the public sector and initiatives from the private sector. The field of AI has enormous potential for optimizing processes and improving medicines and other treatments. Developing countries like Tanzania are critical partners for research, innovation, and development, and their progress is crucial for strengthening global health.

A main recommendation for Germany based on the presented analysis would have been to develop a unified DHS. Given that the FMoH published its first digitization strategy in March 2023 [9], it is advisable to review the previous results and compare them with the goals and measures actually achieved. Developing, implementing, and assessing a DHS requires human capacity, expert knowledge of the current health care system challenges and deployments as well as the legal, social, and ethical framework are needed.

In addition, we recommend the development of step-by-step guidelines and a digital tool for the assessment of national DHSs and their consistency with the WHO’s GSDH, including a recommended benchmarking with a digital maturity model. An important aspect of the guidelines needs to be the ability to capture context-specific elements and adaptations of national DHSs. Having such an assessment tool will enable the comparison of DHSs between countries and provide an overview of the usage of the WHO GSDH as it is a very valuable blueprint for national DHSs.

### Limitations

This study has several limitations. First of all, there is a temporal limitation. DHSs evolve and are updated frequently. The Tanzanian DHS was published 1 year before the WHO GSDH, while different parts of the German strategies were published over several years. Therefore, this work is the state of DHSs in the 2 case study countries as of February 2023, when the analysis was conducted. For instance, the new German Digitalisation Strategy for Health and Care, which was released in March 2023, is not included yet. In addition, there are methodical limitations associated with the policy analysis approach that solely rely on the analysis of policy documents. This may have led to certain aspects not being included in the analysis or being underreported. This applies especially to the case study of Germany with its fragmented strategy across a large number of disparate documents. The level of granularity of the current analysis could be another limitation. Certain aspects of the analysis may have been neglected due to this level of granularity. A more comprehensive evaluation with interviews or surveys with stakeholders could help to provide a deeper understanding.

### Conclusion

The WHO’s GSDH is a valuable blueprint for developing DHSs. Both Tanzania and Germany have developed strategic guidelines aligned with their own national health care priorities. A federal governmental structure, such as in Germany, makes implementing a national DHS more challenging, often leading to many different strategic approaches and priorities. The extremely rapid development and advancement of emerging technologies is a challenge when their development outpaces the speed at which strategies are adapted and implemented, potentially leading to uncoordinated development. Countries need to develop broad DHSs that guide the future development of the DH ecosystem. These strategies need to include frameworks to support the implementation of new technologies to ensure that these technologies are strategically aligned and revised regularly to ensure alignment with new developments and needs.

For the policy assessment of DHSs, in-depth knowledge of the respective country, its health care needs, and health care system structure is needed. Additional data collection, for example, through interviews and surveys with stakeholders, is needed in addition to document reviews to conduct a holistic assessment of a country’s DHS.

## Appendix

Multimedia Appendix 1 Detailed breakdown of the results of the policy comparison by aspect.

Multimedia Appendix 2 Overview of the policy documents mentioned in the methods section with web links.

Multimedia Appendix 3 Evaluation of the strategies of Tanzania and Germany in term of their fulfillment of the 4 dimensions of WHO strategy.

## Abbreviations

AI: artificial intelligence
DH: digital health
DHS: digital health strategy
DiGA: digital health application (digitale gesundheitsanwendungen)
FMoH: Federal Ministry of Health
GSDH: Global Strategy on Digital Health 2020-2025
ICT: information and communication technology
TI: telematics infrastructure
WHO: World Health Organization

## Data Availability

The data sets generated or analyzed during this study are available from the corresponding author upon reasonable request.
