# Supplementary material for: Tanzania’s and Germany’s Digital Health Strategies and Their Consistency With the World Health Organization’s Global Strategy on Digital Health 2020-2025: Comparative Policy Analysis
Source: J Med Internet Res. 2024 Mar 18;26:e52150. doi: 10.2196/52150 (PMC10985601; doi:10.2196/52150)
Supplement: Multimedia Appendix 1 [file jmir_v26i1e52150_app1.docx]

**Multimedia Appendix 1.** Detailed breakdown of the results of the policy comparison by aspect.

| **Global strategy on digital health 2020-2025 – WHO [4]** | **Digital Health Strategy July 2019 - June 2024 – Tanzania [7]** | **Different Approaches of Digital Health Strategies – Germany (various sources)** |
| --- | --- | --- |
| **1) Collaboration and knowledge transfer** | | |
| **Policy options and actions** |  |  |
| 1) co-create the global strategy on digital health and establish mechanisms for strengthening national digital health strategies and implementing key collaborations on agreed-upon appropriate use of digital technologies to achieve national health and well-being goals; | Knowledge management is only developed at the level of health staff and for the population, e.g., through platforms, e-learning etc.; but no knowledge management for methods, techniques and internationally. | As a member of the European Union, Germany is participating in the design of a global strategy and implementation of digital health through various programmes e.g., framework programme Horizon including [14].  Establishing a European Health Data Space to promote the exchange of health data and interoperability [37]. |
| 2) establish a knowledge management approach to identify and share good practices, knowledge about implementation of new methods and techniques, evidence and lessons learned on digital health across countries and international communities; | The Strategy provide mechanisms for knowledge management through the existing capacity building, and technical working groups, communities of practice, as well as monitoring, evaluation and learning frameworks which allow Analysis of the monitoring reports, best practices, and research.  findings for continuous learning to inform the implementation of this Strategy. | Establishment of a research-compatible data infrastructure according to international standards and at European level [16].  Establishment of a knowledge management and research network as a networking platform for global health (Institution: "German Alliance for Global Health Research” [15]. |
| 3) support countries in establishing information centres for disease surveillance to manage and implement timely decisions during epidemics and other public health emergencies; | 1. Strengthen the disease surveillance and response system.  2. Strengthen integration of eIDSR with related systems in East  African Community Partner States.  3. Strengthen digital solutions for the promotion of safe WASH and  food safety services.  4. Implement digital solutions for tracking and reporting injuries,  emergencies, and disasters. | Based on the constitution, the federal states have enacted their laws within the scope of their legislative competence for police hazard prevention as well as for rescue services, fire protection and disaster control. In addition, a crisis management system has been established{Inneren, 2015 #61}.  In the event of transnational, national danger or damage situations, there is a need to combine many different command and communication structures of the federal government and the federal states into a nationwide crisis management system. |
| 4) align countries and stakeholders to address collectively global, regional, and national challenges and opportunities; identify, manage, and communicate risks; and mitigate threats associated with the use of digital technologies to improve health and enable universal health coverage, the core of the health-related Sustainable Development Goals. | SWOT analysis for the development and implementation of the National Digital Health Strategy 2019-2024 is available; national aspirations are addressed, not global ones | Strengthen health systems and the decentralised, time and location-independent care of individuals. Digital healthcare networks should promote regional and intersectoral, e.g., connection to telematics infrastructure [16]. |
| **Output** | | |
| 1) digital health is prioritised and integrated into health systems at global, regional, and national levels through dedicated bodies and mechanisms for governance; | The main components of the digital health governance structure are based on a national level, but no bodies are responsible for the global level. | There is no national strategy of its own, but  national and initiative/ forum limited until the end of 2022:  - In the innovation forum "Digital Health 2025", the Federal Ministry of Health has given experts from all areas of the healthcare system room for discussion in order to work out together how the opportunities and possibilities of digitalisation can be consistently used for better health care beyond the legislative period [16].  With the strategy, the German government aligns its actions to new challenges, develops its goals further and contributes to the coordination and cooperation of all actors. Implementation Of this strategy will fit into the Federal Government's budgetary and financial policy guidelines [16] . |
| 2) multistakeholder groups are convened regularly to support the appropriate use and scaling up of digital health and innovation to accelerate progress towards health-related Sustainable Development Goals; | The digital health governance structure (there are different digital health committees) provides an avenue to multistakeholder meetings to  ensure that the digital health strategy is in line with national health priorities and that planned activities are properly implemented to achieve the desired outcomes. | E-health initiative to hold regular meetings to identify implementation hurdles for the establishment of digital applications, such as telemedicine, and to develop packages of measures to reduce these hurdles Participants include all self-government organisations involved in standard care, but also the relevant business associations whose member companies offer information and communication technologies and IT solutions for the health sector.  Zukunftsregion Digitale Gesundheit (ZDG) is an initiative of the Federal Ministry of Health that is scheduled to run until the end of 2022 (Involvement of user perspectives). |
| 3) information centres for disease surveillance are established or strengthened at national, regional, and global levels. | Various systems exist, e.g., eIDSR,  WASH, National Sanitation Management Information System. | Act on the Modernisation of the Epidemiological Surveillance of Communicable Diseases, e.g. Outbreaks of infectious diseases are reported to the Robert Koch Institute.  With the One Health approach against infectious diseases (to combat antimicrobial  resistance), data is exchanged internationally. |
| **2) Advance the implementation of national digital health strategies** | | |
| **Policy options and actions** | | |
| 1) stimulate and support every country to adopt or review, own, and strengthen its national digital health strategy in a way that enhances the level of country maturity regarding digital health to achieve positive health outcomes in line with the national health plans, updated norms and standards recommendations, and universal health coverage; | Strategy revised with new paper. | Due to the funding system in Germany, each federal state has its own Ministry of Health at the federal level and can, therefore, enact its own laws and rules. However, overarching legislation such as TI connectivity can bring the federal states into line and encourage them to rethink their national strategy, e.g., connection to the TI.  The Conference of Health Ministers takes place once a year. |
| 2) ensure that institutions, decision-makers, and personnel involved in the provision of health care services and all end-user communities and beneficiary populations are adequately engaged in the design and development phases; | Digitisation of health services at the level of health institutions.  Digitisation of community health services.  Establish an inventory of digital health initiatives to track and coordinate.  Stakeholder engagement and involvement of health workers in digital transformation efforts. | Not addressed |
| 3) facilitate a systematic engagement of all relevant stakeholders in the realisation of the vision and its strategic objectives as part of an integrated digital health ecosystem at the national level; | Part of the implementation involves stakeholders (insurance companies, institutions, customers, etc.) through digital solutions.  Vision: Better health outcomes through a digitally enabled health system. | Establishment of initiatives and stakeholder meetings but lack user and patient level, e.g., no mandatory collection of patient-reported outcome measures. |
| 4) define a national digital health architecture blueprint or road map, adopt open-source health data standards, and aim for reusable systems or assets, including interoperability of health information systems both at national and international levels in order to establish an innovative integration of different digital technologies using shared services, ensuring data are of good and comparable quality; | 1. Finalise and institutionalise the Tanzania Health Enterprise Architecture.  2. Strengthen the use of data, application, and technology standards  3. Implement terminology services for standardised health  terminologies, codes, data elements, and value sets.  4. Strengthen interoperability | Since 2016, a formal roadmap for establishing digital health has existed in Germany at the federal level. It focuses on using the telematics infrastructure as the digital infrastructure for the health care system. It places patient benefits and the highest level of data protection at the centre. A concrete strategy to support technical and semantic interoperability is not yet apparent. As a step in this direction can be seen as the activation of Gematik's Vesta interoperability directory. It may be considered as a step in this direction. There are no comprehensive binding target formulations, guidelines, or deadlines for a digital health system . |
| 5) adopt legal and ethical frameworks for assuring patient safety, data security, appropriate use and ownership of health data, privacy data recoverability, as well as protection of intellectual property rights; | Improve the legal and regulatory framework to ensure client.  safety, data security, confidentiality, and privacy. | Legal: Health data enjoy stringent protection in the Regulation as special personal data (cf. Art. 9 DSGVO).    Patient Data Protection Act .  Global Action Plan for Patient Safety 2021-2030.  Ethics Commission, as well as ELSA Research of the Federal Ministry of Education and Research, deals with the ethical, legal, and social aspects of the life sciences. |
| 6) identify and promote sustainable financing models supporting digital health development and sharing learning to inform future products and services. This is especially important in artificial intelligence, including machine learning, implementation, integration, and maintenance, including economic incentives and | Strengthen digital solutions for funding management within institutions, e.g., for auditing or electronic revenue collection like Government ePayment Gateway, Planning, financial management, accountability and reporting systems.  There is also a national digital health investment roadmap; only rough. | Various instruments and models exist but are not transparent (e.g., only a few know about high-tech strategy promotion) [24].  Another problem is sustainability. Projects like the Health Innovation Hub are only temporary. |
| 7) design, implement and monitor a change management plan to support conducive organisational behaviour surrounding newly digitised health processes and practices. | Governance is developing a plan to manage change. | Not addressed |
| **Output:** | | |
| 1) a national digital health strategy or equivalent strategic framework exists, is integrated in the national health strategy, and is actively used to guide development and accelerate progress towards the health-related targets of the Sustainable Development Goals and in the context of digital transformation of health systems; and | Strategy for digital health exists, and it is aligned with the national health sector strategic plan and national health policy. | Activities are aligned with a target and implementation vision for the coming years until 2025 and harmonised with other processes, such as the roadmap for the development and implementation of innovative e-health solutions. The focus is on overcoming sectoral boundaries [16].  However, a national digital health strategy with binding goals and guidelines is missing. Only individual applications are regulated by the e-health law. |
| 2) a dynamic digital health maturity model assessment to guide prioritization of national investment in digital health is made in support of primary health care and universal health coverage. | Priority setting is already fulfilled through strategy. The strategic priorities were primarily derived from the National Health Policy 2019 and through a rigorous consultation process with key stakeholders in the public and private health sectors as well as related sectors e.g. ICT. The Strategy was also informed by a landscape assessment and review of the implementation of the previous strategy and secondary research on best practices in the field.  Data use as a Quality aspect: The government can efficiently assess and improve the quality of health services. | Digital Health 2025 is an important step towards taking stock and defining goals. Perspectives of the end user in the foreground. However, there is no clear prioritisation. |
| **3) Strengthen governance for digital health at global, regional, and national levels** | | |
| **Policy options and actions** | | |
| 1) strengthen governance of digital health at national and international levels by leveraging existing structures and as appropriate creating sustainable and robust governance structures, including regulatory frameworks, and the capacity for the implementation of evidence-based and proven digital health solutions at global and national levels; | Strengthen governance structures to enable effective coordination, management oversight and implementation of digital health initiatives across the health sector.  Develop guidelines for the implementation of digital health, establish a legal and regulatory framework for digital health.  Develop a resource mobilisation plan to ensure successful implementation of the strategy. | Developments available e.g., since 2019, the Federal Ministry of Health retains 51% of the shares in gematik. This is responsible for TI in Germany.  Federal Ministry of Health (BMG) has passed important framework conditions and laws in recent years, e.g., Health IT Interoperability Governance Ordinance (GIGV). |
| 2) coordinate investments in evidence-based approaches to assess promote and disseminate new and innovative health technologies for national scaled digital health programmes using a person-centred approach to facilitate actions and investments based on informed decisions; | Evidence-based decision-making is addressed in Priority 5 and 6 (investment in new health care architecture to use digital technologies and dissemination of qualitative data for evidence-based action). | DiGAs as evidence-based digital health solutions with high requirements for evidence studies [29]. |
| 3) promote and facilitate digital health competencies in the education and training curricula of all health professionals and allied workers; and | Introduction of digital platforms for networking of health professionals.  Introduce e-learning and knowledge management platforms for continuous professional development.  Strengthen continuous professional development programmes for health workers in the use of data. Include aspects of data use in education and training curricula. | Digital continuing education in the health sector: The Federal Ministry of Education and Research launches a funding guideline for the development and testing of digital education and training measures for health professions.  Criticism: started in 2018, no changes yet, no concepts for the future available from the government. |
| 4) promote capacity-building for leaders of public health authorities, affiliated agencies, and policymakers to take informed decisions to support digital health investments. | Strengthen national, regional, and international collaborations as a vehicle for capacity building on emerging digital health innovations. Strengthen different Communities of Practice for digital health. | Not addressed |
| **Output:** | | |
| 1) governance exists, in accordance with Secretariat-led development of regulatory framework, to agree on global appropriate use of health data and on concepts such as health data as a global public good and to outline principles of equitable data-sharing principles for research, consistent metadata and definitions, artificial intelligence and data analytics, and primary and secondary use of data; | Data analysis tools and indicators must be developed. | Legal framework and responsibilities are not yet in place, but e.g., voluntary data donation should be regulated with legal clarity [16].  Promote AI by developing evaluation standards for algorithm-based applications. Adaptation of the legal framework nationally and to technological advances is planned [16]. |
| 2) a voluntary guideline on global interoperability standards for digital health is developed in collaboration with stakeholders and adopted, that a) tries to build upon results already broadly achieved, b) includes a list of commonly agreed use cases for the public health care sector, its functional requirements and a set of functional and technical specifications, standards, semantics and profiles derived thereof, c) defines requirements for a sound legal and regulatory framework with clearly defined roles for data governance and d) encompasses political leadership regarding public investment, procurement and standardization to create an interoperable digital health ecosystem at the national and international levels; | Governance enforces compliance with digital health guidelines and standards, but it does not say whether they are international interoperability standards. | Not addressed |
| 3) global guidance on planning, development and use of digital hospitals, digital clinical trials and digital therapeutics is developed; and | Covered under the Tanzania Health Enterprise architecture. As well as a priority to develop legislation, regulations, and guidelines for ensuring client safety, health data security, confidentiality, and privacy. | No global guidelines, only e.g., laws such as the Hospital Future Act.  Topics such as e-trial management systems are not addressed. |
| 4) a set of recommendations is developed for pseudonymization and anonymization of health data. | Covered under the Tanzania Health Enterprise architecture. As well as a priority to develop legislation, regulations, and guidelines for ensuring client safety, health data security, confidentiality, and privacy. | Data protection incl. pseudonymization and anonymisation is an important priority in Germany; various working groups are dealing with this e.g., GMDS working group or Data Protection and IT Security in the Healthcare Sector (DIG). |
| **4) Advocate people-centred health systems that are enabled by digital health** | | |
| **Policy options and actions** | | |
| 1) place people at the centre of digital health through the appropriate health data ownership, adoption and use of digital health technologies and development of appropriate literacy; the focus will cover not only patients, families, and communities but also health workers; | Improving digital skills e.g., health workers to provide specialised care, but ownership of data is not highlighted. | The Federal Government wants to promote e.g., personalised treatment approaches in all important disease areas and Germany are raising awareness nationwide with e.g. electronic patient files that the patient is at the centre, because the patient has the supremacy with regard to data [32].   gesund.bund. de Portal for digital health literacy [31].   More digital helpers for those in need of care, more telemedicine, and a good digital infrastructure - these are the goals of the Act on the Digital Modernisation of Care and Nursing. |
| 2) develop approaches to the management of health at the population level through digital health applications that move health and well-being from reactive-care models to active community-based models, and reduce the burden of data collection from front-line workers by reorienting reporting-based tools into service delivery tools; | Digital solutions to manage at population level is addressed on priority 2. | Continuously think and design digital care of the future, for example digital prevention programmes and holistic care offers.  Act on the digital modernisation of care and nursing from 2021; Digital nursing applications as relief and digital documentation, connection to the TI. |
| 3) establish monitoring and evaluation models to facilitate monitoring the contribution of digital systems to health system processes, health workforce processes, and individual and community health needs; | Various tools are available but no indication of exact assessment models. | gematik TI Dashbaord; processes of the health workforce and the individual and community health needs are not shown [33]. |
| 4) strengthen gender equality and health equity approaches and accessibility for people with disabilities to promote inclusive digital society with enhanced digital health skills. When planning and prioritizing digital health interventions, relevant factors of inequality should be assessed to ensure that the introduction of digital health technologies does not aggravate these (“do no harm”) and that access for specific population groups is guaranteed. In addition, the specific potential of digital technologies to promote health equity should be leveraged. Designed properly, digital solutions can propel inclusiveness as digital connectivity can transcend physical barriers; | Equal opportunities are addressed on priority 3 (Telemedicine access to mitigate physical barriers), but unclear how older people are involved in the technology process. Change and adoption  Capacity building and adopting the Principles for Digital Development.  Inclusion, gender is not addressed.  Guiding principles cater to address, e.g. mapping of the end-to-end business process and user-centric and data-drive | Not addressed |
| 5) implement mechanisms for more effective public participation and transparency in national and international digital health decision-making processes, such as through international consultation processes or a stakeholder forum; | Public is informed but not involved in decision making but feedback from clients is collected and considered. | Innovation Forum "Digital Health 2025", the BMG has involved numerous experts from all areas of the health care system in a stakeholder process in 2019; national decisions and only experts from the health care sector are involved [16]. |
| 6) develop digital health training or Massive Open Online Courses to improve digital health literacy; and | Implementation of e-learning and knowledge management platforms for continuous professional development and awareness, but no trainings for public. | National health portal for education [31].  DGV obliges health insurance companies to offer digital health competences. |
| 7) create an international communication campaign to sensitize people to the benefits of digital health solutions and the use of their data for public interest research, and thereby promote the vision of people being actors of innovation. | Research is not explicitly mentioned and therefore no data, but the population is made aware of the advantages of health solutions through platforms for education. | International campaign not addressed, but Germany raises awareness nationwide, e.g., with electronic patient files, that the patient is at the centre, because he or she has the supremacy regarding the data. |
| **Output:** | | |
| 1) Improved digital health literacy in using and understanding digital health technologies and systems as well as health data is prioritized, and the validated tools are accessible by all populations; | The government can effectively monitor the performance of the health system and the quality of health services provided efficiently.  The government can efficiently assess and improve the quality of health care.  Clients have access to IEC in the health sector to promote healthier behaviour. The health sector can systematically use interactive digital platforms for health education.  The health sector can provide IEC on health using interactive digital platforms. Health facilities and insurance service providers can process insurance claims more efficiently, leading to an increase in financial resources for health facilities. | Develop a communication strategy for digital health applications and for digital health in the broader sense.  Focus on the acceptance of the population, to strengthen a deeper understanding of digital health literacy of patients, to build and expand a national health portal (search for understandable and scientifically sound health information) [16].  Introduction of the National Health Portal [31]. |
| 2) a framework allowing individual feedback in validating the performance of digital health tools and services, diffusion of increasing digital health demand is implemented and used; | No overarching platform or framework to collect feedback but, health workers at all levels can efficiently deliver quality health care to improve customer experience.  The health sector can capture and respond to client feedback and needs. | Not addressed |
| 3) global minimum health data standards for prioritized digital health technologies and processes are established, adopted, and applied at national level; and | Health enterprise architecture has been developed to guide digital health and health data standardization. Interoperability and standards are listed nationally | E.g., SNOMED CT - national licence for this international terminology [16].  DSGVO shows as an example that Germany is also oriented towards the EU and thus thinks more internationally than nationally.  In the German government's strategy for global health, various approaches to e.g., international standards can be found [13]. |
