# Supplementary material for: Tanzania’s and Germany’s Digital Health Strategies and Their Consistency With the World Health Organization’s Global Strategy on Digital Health 2020-2025: Comparative Policy Analysis
Source: J Med Internet Res. 2024 Mar 18;26:e52150. doi: 10.2196/52150 (PMC10985601; doi:10.2196/52150)
Supplement: Multimedia Appendix 2 [file jmir_v26i1e52150_app2.docx]

# APPENDIX 2

| **Ref** | **Document Title and URL** |
| --- | --- |
| [4] | Global strategy on digital health 2020-2025 - WHO  https://www.who.int/publications/i/item/9789240020924 |
| [7] | Tanzania National eHealth Strategy 2013 – 2018  https://health.eac.int/file-download/download/public/228 |
| [8] | Digital Health Strategy July 2019 - June 2024 - Tanzania  https://media.path.org/documents/Tanzania_Digital_Health_Strategy_2019_-2024.pdf |
| [9] | DIGITAL TOGETHER Germany’s Digitalisation Strategy for Health and Care  https://www.bundesgesundheitsministerium.de/fileadmin/Dateien/3_Downloads/D/Digitalisierungsstrategie/Germany_s_Digitalisation_Strategy_for_Health_and_Care.pdf |
| [13] | Strategie der Bundesregierung zur globalen Gesundheit https://www.bundesgesundheitsministerium.de/fileadmin/Dateien/5_Publikationen/Gesundheit/Broschueren/GlobaleGesundheitsstrategie_Web.pdf |
| [14] | Horizon Europe Program  https://www.horizont-europa.de/de/Programm-1710.html |
| [15] | German Alliance for Global Health Research (GLOHRA)  https://globalhealth.de |
| [16] | Digitale Gesundheit 2025.  https://www.bundesgesundheitsministerium.de/fileadmin/Dateien/5_Publikationen/Gesundheit/Broschueren/BMG_Digitale_Gesundheit_2025_Broschuere_barr.pdf |
| [17] | Gesetz für sichere digitale Kommunikation und Anwendungen im Gesundheitssektor  https://www.bgbl.de/xaver/bgbl/start.xav?startbk=Bundesanzeiger_BGBl&start=//*%5B@attr_id=%27bgbl115s2408.pdf%27%5D#__bgbl__%2F%2F*%5B%40attr_id%3D%27bgbl115s2408.pdf%27%5D__1652087696139 |
| [18] | Gesundheitsministerkonferenz  <https://www.gmkonline.de/Die-GMK.html>. |
| [19] | Roadmap Digitale Gesundheit  https://www.bertelsmann-stiftung.de/de/publikationen/publikation/did/roadmap-digitale-gesundheit |
| [20] | Gesundheits-IT Interoperabilität Governance Verordnung  <https://www.bgbl.de/xaver/bgbl/start.xav?startbk=Bundesanzeiger_BGBl&start=//*%5b@attr_id=%27bgbl121s4634.pdf%27%5d#__bgbl__%2F%2F*%5B%40attr_id%3D%27bgbl121s4634.pdf%27%5D__1652088273525> |
| [21] | Daten helfen heilen  https://www.bmbf.de/SharedDocs/Publikationen/de/bmbf/6/23360_Daten_helfen_heilen.pdf?__blob=publicationFile&v=3 |
| [22] | Datenschutz und IT-Sicherheit im Gesundheitssektor  https://www.gesundheitsdatenschutz.org/download/einwilligung_2021.pdf |
| [23] | Gesetzes zur digitalen Modernisierung von Versorgung und Pflege (Digitale-Versorgung-und-Pflege-Modernisierungs-Gesetz – DVPMG)  https://www.bundesgesundheitsministerium.de/fileadmin/Dateien/3_Downloads/Gesetze_und_Verordnungen/GuV/D/DVPMG_BT_bf.pdf |
| [24] | Hightech-Strategie 2025  <https://www.bmbf.de/bmbf/de/forschung/hightech-strategie-2025/hightech-strategie-2025_node.html> |
| [25] | European Health Data Space  https://health.ec.europa.eu/publications/proposal-regulation-european-health-data-space_en |
| [26] | Tanzania Health Enterprise Architecture  <https://media.path.org/documents/Tanzania_Enterprise_Architecture.pdf?_gl=1*vsfcix*_gcl_au*MTI5NjIxODA0NS4xNzAxMTc1Nzc4*_ga*MTkwMjYzNDA5Mi4xNzAxMTc1Nzc4*_ga_YBSE7ZKDQM*MTcw> |
| [27] | Tanzania Digital Health Investment Road Map 2017-2023  https://media.path.org/documents/Tanzania_Digital_Health_Investment_Road_Map.2017_to_2023.pdf |
