# Supplementary figures and images for: Tanzania’s and Germany’s Digital Health Strategies and Their Consistency With the World Health Organization’s Global Strategy on Digital Health 2020-2025: Comparative Policy Analysis
Source: J Med Internet Res. 2024 Mar 18;26:e52150. doi: 10.2196/52150 (PMC10985601; doi:10.2196/52150)

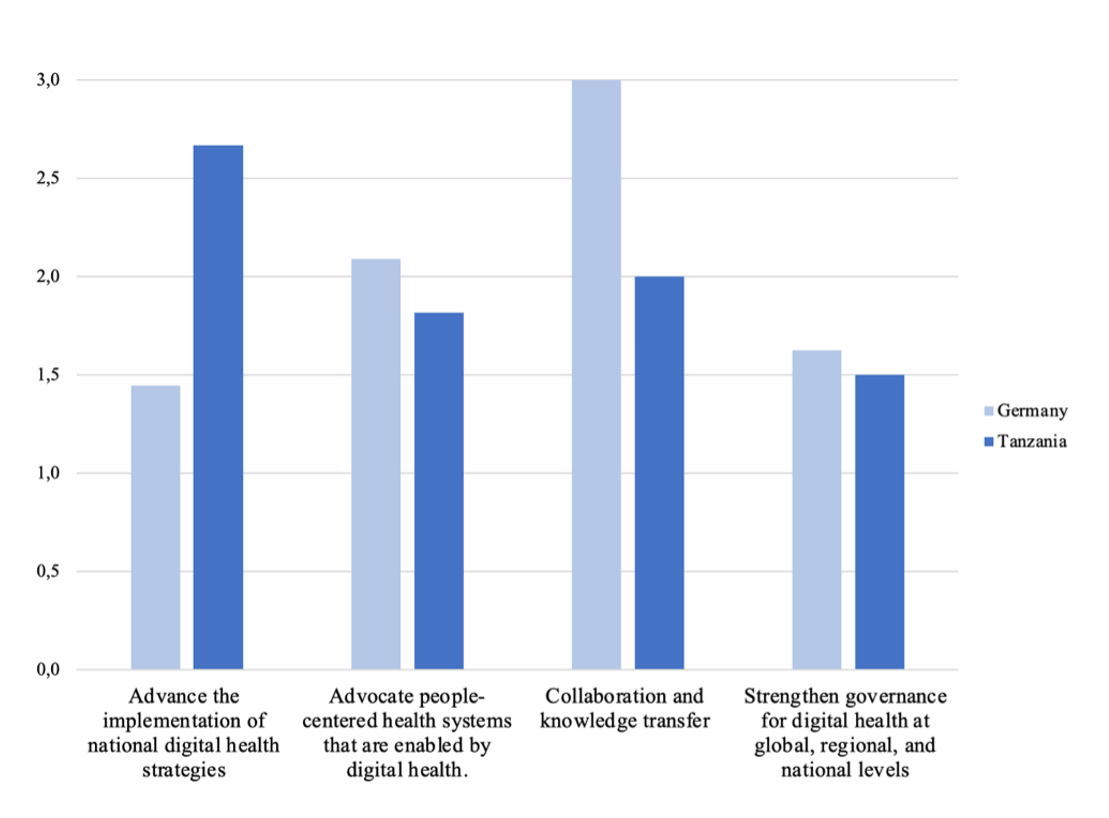

Supplement: Multimedia Appendix 3 [file jmir_v26i1e52150_app3.png]
